# Hepatobiliary disorders associated with TNF-α inhibitors: a pharmacovigilance analysis of FAERS and JADER

**DOI:** 10.3389/fimmu.2025.1739631

**Published:** 2026-01-12

**Authors:** Hongyu Chen, Shirui Jiang, Jingyu Wang, Ailin Zhang, Liqin Zhu

**Affiliations:** 1 First Central Hospital of Tianjin Medical University, Tianjin, China; 2 Tianjin University of Traditional Chinese Medicine, Tianjin, China; 3 Department of Pharmacy, Tianjin First Central Hospital, Tianjin, China

**Keywords:** disproportionality analysis, hepatobiliary disorders, pharmacovigilance, rheumatology, risk factors

## Abstract

**Introduction:**

Tumor necrosis factor-a (TNF-a) inhibitors are widely used for immune-mediated diseases. Their systemic adverse events (AEs) have become increasingly prominent. The hepatobiliary risks of different TNF-a inhibitors were compared in this paper to serve for their screening in clinics.

**Methods:**

We analyzed the U.S. Food and Drug Administration Adverse Event Reporting System (FAERS) data and Japanese Adverse Drug Event Report Database (JADER) data for five TNF-a inhibitors (adalimumab, etanercept, infliximab, certolizumab pegol, golimumab) listed as primary suspects. Disproportionality analyses identified hepatobiliary safety signals. Logistic regression assessed risk factors.

**Results:**

Across FAERS and JADER, adalimumab showed a positive hepatobiliary signal [ROR: 1.42 (1.39, 1.45) in FAERS and 1.39 (1.20, 1.62) in JADER].For adalimumab, risk was higher in men [OR: 1.225 (1.082, 1.386) in FAERS and 2.378 (1.517, 3.726) in JADER] and with higher dose [OR: 1.073 (1.049, 1.098) in FAERS], higher age [OR: 1.168 (1.032, 1.321) in JADER], greater weight [OR: 1.363 (1.192, 1.557) in JADER], and several co-reported categories which included skin and subcutaneous tissue disorders [OR: 1.247 (1.084, 1.434) in FAERS and 2.432 (1.100, 5.437) in JADER].

**Conclusion:**

The robust signal between adalimumab and hepatobiliary adverse events was observed in our study. Male sex and skin and subcutaneous tissue disorders were regarded as consistent independent risk factors. The factors of drug dose, body weight, and additional comorbidity clusters could also require attention in clinics because these factors were obviously observed in certain single databases.

## 1 Introduction

Tumor necrosis factor-α (TNF-α) inhibitors have become a cornerstone in the management of immune-mediated diseases. These agents exert their effects by antagonizing TNF-α, a pro-inflammatory cytokine that plays a central role in immune activation and inflammation. TNF-α inhibitors have been widely utilized in rheumatology, gastroenterology, and dermatology by alleviating inflammation and improving disease symptoms (1). To date, adalimumab, etanercept, infliximab, certolizumab pegol, and golimumab have been approved by the U.S. Food and Drug Administration (FDA) for use in humans (2). These biologics are indicated for a broad spectrum of immune-mediated conditions, including rheumatoid arthritis, Crohn’s disease, ulcerative colitis, and psoriasis (3–6). According to current guidelines from the European League Against Rheumatism (EULAR) and the American College of Rheumatology (ACR), TNF-α inhibitors are recommended as first-line biologic agents for patients inadequately controlled by conventional therapies (7, 8).

Despite their clinical efficacy, TNF-α inhibitors are associated with a range of adverse effects. These adverse events (AEs) span a wide spectrum, from mild injection-site reactions to serious infections and malignancies (9). Rare but severe AEs include cardiovascular events, intestinal obstruction, immune deficiencies, and hepatobiliary disorders (10).

Among these, hepatobiliary disorders constitute a rare but clinically important category of AEs. Several studies and case reports have described hepatobiliary AEs, including drug-induced hepatitis, autoimmune hepatitis, and cholestasis (11–14). For instance, infliximab has been linked to severe liver injury requiring liver transplantation, while adalimumab and etanercept have been associated with elevated liver enzymes. Case series and a pharmacovigilance database study have reported autoimmune hepatitis and other hepatobiliary events with anti-TNFα agents (14, 15); a systematic review has also summarized biologic-related liver injury across agents (13), but head-to-head comparisons among TNF inhibitors remain scarce. Moreover, whether hepatobiliary reporting patterns are consistent across different spontaneous reporting systems (e.g., FAERS vs JADER) remains unclear.

Therefore, this study aimed to systematically evaluate hepatobiliary AEs associated with TNF-α inhibitors using the FAERS (2004–2024) and JADER (2004–2025) databases, providing a cross-database hepatobiliary disproportionality comparison across TNF-α inhibitors, and to explore potential risk factors for the hepatobiliary AE signal-positive TNF-α inhibitor. The findings may help inform clinical decision-making and support risk–benefit assessments for TNF-α inhibitor therapy.

## 2 Methods

### 2.1 Data source

This pharmacovigilance analysis utilized data from FAERS and JADER. FAERS is maintained by the U.S. FDA. It includes seven datasets: demographics/administrative (DEMO), drug information (DRUG), adverse events (REAC), outcomes (OUTC), report sources (RPSR), therapy start/end dates (THER), and indications (INDI) (16, 17). JADER is maintained by Japan’s PMDA. It includes four core datasets: DEMO, DRUG, REAC, and primary disease (HIST). Adverse events were coded with MedDRA Preferred Terms (PTs), version 27.1.

### 2.2 Data preparation

Reports related to TNF-α inhibitors were collected from FAERS for the period spanning Jan 01, 2004 to Dec 31, 2024, and in JADER from Jan 01, 2004 to Sep 30, 2025, including adalimumab, etanercept, infliximab, certolizumab pegol, and golimumab. Only cases in which TNF-α inhibitors were identified as the suspected drugs were included. Reports in which the TNF-α inhibitors were identified as Primary Suspect (PS) drugs in the FAERS database were included for analysis. In the JADER database, only records in which the TNF-α inhibitors were classified as suspected drugs were extracted.

All analyses were performed using R (version 4.4.2), Microsoft Excel, and IBM SPSS Statistics (version 26). Records without a case number, suspected drugs, or identified adverse reactions were excluded. In FAERS, CASEID and PRIMARYID are respectively unique case identifier and unique report identifier. FDADT denotes date of FDA receipt. For duplicate reports, the most recent record was retained according to FDA recommendations. (18). The filtering process was based on CASEID, FDADT, and PRIMARYID, with criteria specified as follows: (1)For records sharing the same CASEID, retain only the record with the maximum FDADT. (2)For records with identical CASEID and FDADT, retain only the record with the maximum PRIMARYID. Similarly, reports were extracted from the Japanese Adverse Drug Event Report (JADER) database. Data modules including DEMO (demographics), DRUG, REAC, and HIST were linked by case ID. Duplicate reports were excluded following PMDA guidance. AEs were coded using MedDRA, and hepatobiliary AEs were identified at the System Organ Class (SOC) level (“Hepatobiliary disorders”). All PTs under this SOC were included.

### 2.3 Data analysis

A 2×2 contingency table was constructed for the SOC “Hepatobiliary disorders” to evaluate the relationship between individual TNF-α inhibitors and hepatobiliary AEs (as shown in **Supplementary Table S1**) (19).

Reporting Odds Ratios (RORs) and 95% confidence intervals (CIs) were calculated (20). The Bayesian Confidence Propagation Neural Network (BCPNN) was used in parallel as a complementary disproportionality method (21, 22). Parallel disproportionality analyses (ROR, IC) were performed in both FAERS and JADER datasets independently. Signals were compared across the two databases to evaluate consistency and robustness. Comparison were conducted between the target TNF-α inhibitor and the remaining TNF-α inhibitors in the main analysis. All statistical analyses were conducted via R version 4.4.2 and SPSS Statistics 26.The specific formulas and thresholds can be found in **Supplementary Table S2** (23).

To handle missingness, complete cases were used for primary analysis. Records with available age, sex, body weight, dose, and comorbidity information were retained and constituted the analytic dataset. Separate multivariable logistic regression models were then fitted in FAERS and JADER, with hepatobiliary adverse event (yes/no) as the dependent variable. Candidate risk factors included age, sex, body weight, adalimumab dose, and MedDRA-based comorbidity categories. Continuous predictors were scaled per 10 units (dose per 10 mg, age per 10 years, weight per 10 kg) to improve interpretability. A forward stepwise selection procedure was used to enter variables sequentially, yielding the final model in each database. Results are presented as odds ratios (ORs) with 95% confidence intervals. Predictive performance was not evaluated because the model was intended for risk factor association analyses rather than prediction.

## 3 Results

### 3.1 Hepatobiliary AEs among users of TNF-α inhibitors in the FAERS and JADER

**Figure 1:**
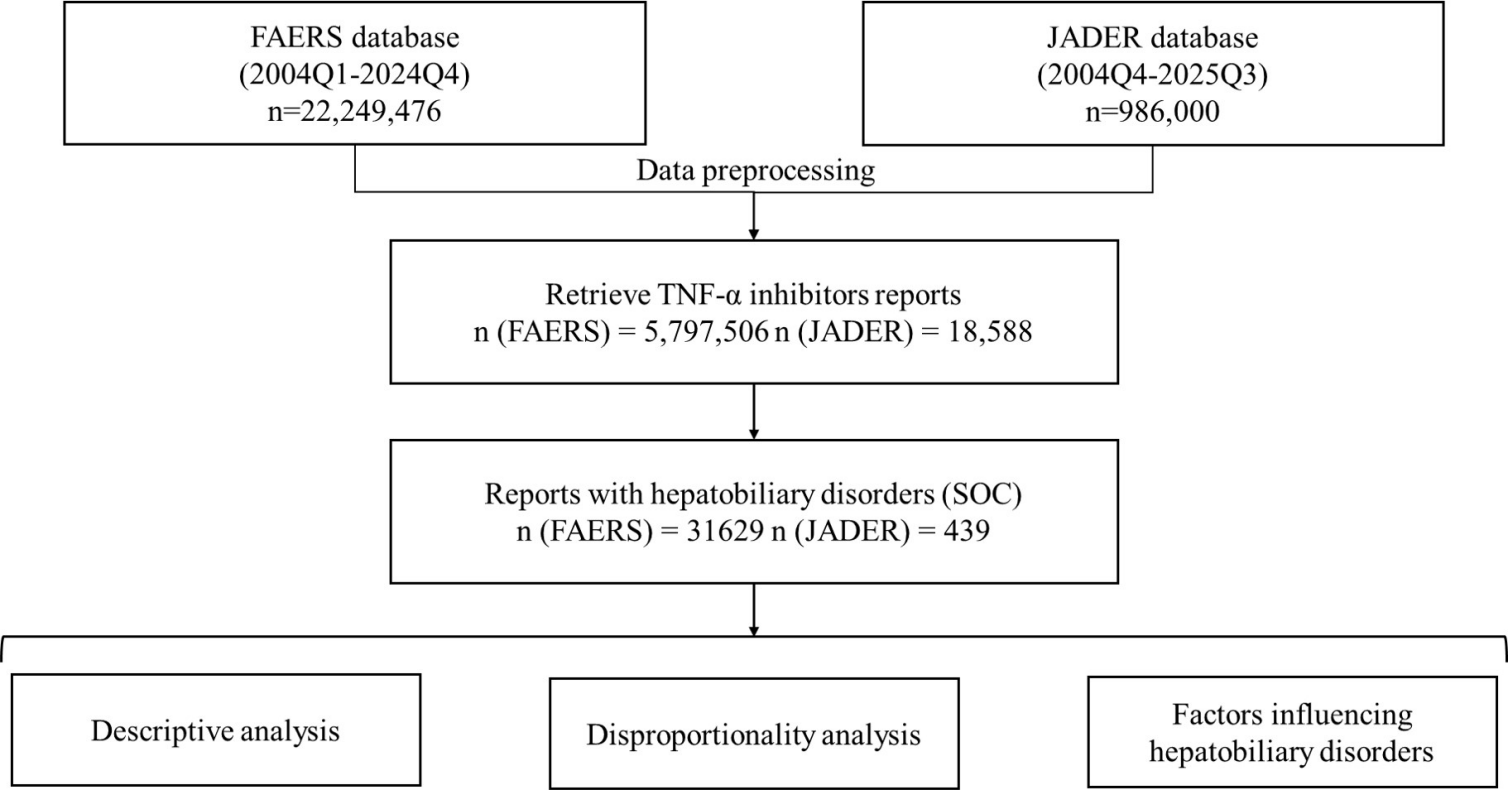
Flow chart illustrating the data selection and analysis process for hepatobiliary AEs induced by TNF-α inhibitors based on the FAERS and JADER.

The occurrence of hepatobiliary AEs associated with TNF-α inhibitors were reported in both the FAERS and JADER databases. This analysis has yielded a comprehensive dataset of hepatobiliary AEs induced by TNF-α inhibitors (**Figure 1**). After data cleaning and removal of duplicate records, a total of 19,083 hepatobiliary AE cases related to TNF-α inhibitors were identified in FAERS. In the FAERS data, adalimumab accounted for the largest number of hepatobiliary AE cases (9501, 49.79%), followed by infliximab (3979, 20.85%), etanercept (3949, 20.69%), certolizumab pegol (1012, 5.30%), and golimumab (642, 3.36%).In the JADER database, a total of 409 hepatobiliary AE cases related to TNF-α inhibitors were identified. Etanercept accounted for 120 cases (29.34%), followed by infliximab (116; 28.36%), adalimumab (96; 23.47%), golimumab (54; 13.20%), and certolizumab pegol (23; 5.62%). Although the relative proportions differed slightly between the two databases, both showed that adalimumab, infliximab, and etanercept were the most frequently reported agents associated with hepatobiliary disorders.

Demographic features of patients with hepatobiliary AEs reported in the FAERS and JADER databases were described in **Table 1**. A total of 19,083 cases were retrieved from FAERS and 409 from JADER. Among cases with available sex information, females accounted for 66.27% in FAERS and 60.68% in JADER, indicating a similar gender distribution between the two databases. Age information was available for 12,485 FAERS cases and 388 JADER cases. The median age of patients in FAERS was 54 years (interquartile range 43–64 years), while most JADER cases were between 60 and 79 years old, suggesting that the Japanese reports involved relatively older patients. Weight data were less frequently available (6,560 in FAERS and 233 in JADER). The median body weight in FAERS was 75 kg (IQR 62–91 kg). Compared with FAERS, the JADER dataset showed a higher proportion of patients in the 40–69 kg range, reflecting population and reporting differences.

**Table 1:** Demographic characteristics of unique cases (after deduplication) involving TNF-α inhibitor–associated hepatobiliary AEs in the FAERS and JADER database.

Characteristics	FAERS No. (%)	JADER No. (%)
Number of unique cases (after deduplication)	19083	409
Gender		
Data available	17300	396
Female	11464 (66.27%)	241 (60.86%)
Male	5836 (33.73%)	155 (39.14%)
Age (years)		
Data available	12485	388
<20	478 (3.83%)	7 (1.80%)
20-29	734 (5.88%)	17 (6.19%)
30-39	1319 (10.56%)	26 (6.70%)
40-49	2286 (18.31%)	46 (11.86%)
50-59	3186 (25.52%)	85 (21.91%)
60-69	2895 (23.19%)	101 (26.03%)
70-79	1334 (10.68%)	85 (21.91%)
≥80	253 (2.03%)	21 (5.41%)
Median (IQR)	54 (43-64)	not applicable
Weight (kg)		
Data available	6560	233
<30	90 (1.37%)	2 (0.86%)
30-39	72 (1.1%)	22 (9.44%)
40-49	363 (5.53%)	44 (18.88%)
50-59	831 (12.67%)	71 (30.47%)
60-69	1216 (18.54%)	60 (25.75%)
70-79	1215 (18.52%)	23 (9.87%)
80-89	970 (14.79%)	7 (3.00%)
90-99	788 (12.01%)	2 (0.86%)
100-109	417 (6.36%)	1 (0.43%)
≥110	598 (9.12%)	1 (0.43%)
Median (IQR)	75 (62-91)	not applicable
Drug used		
Adalimumab	9501 (49.79%)	96 (23.47%)
Certolizumab pegol	1012 (5.30%)	23 (5.62%)
Etanercept	3949 (20.69%)	120 (29.34%)
Golimumab	642 (3.36%)	54 (13.20%)
Infliximab	3979 (20.85%)	116 (28.36%)

N/A, not applicable.

Median (IQR) is not applicable for JADER due to non-continuous coding.

### 3.2 Disproportionality analysis for TNF-α inhibitors

A single case can contribute multiple paired records of PT. A total of 31,629 hepatobiliary AE reports from FAERS and 439 from JADER were included in the disproportionality analysis (**Table 2**). In both databases, adalimumab exhibited significant positive signals, with RORs of 1.42 (95% CI 1.39–1.45 in FAERS and 1.39 (95% CI 1.20–1.62) in JADER, and corresponding IC025 values above zero (0.25 and 0.14, respectively), indicating a consistent association between adalimumab and hepatobiliary AEs. Golimumab also demonstrated a positive signal in FAERS (ROR = 1.18, 95% CI 1.10–1.26; IC025 = 0.13) but not in JADER (ROR = 0.92, 95% CI 0.80–1.06; IC025 = −0.28), suggesting inconsistency across data sources. Etanercept, infliximab and certolizumab pegol did not show significant disproportionality signals. Overall, adalimumab showed the most robust and consistent association with hepatobiliary disorders across both pharmacovigilance databases, while findings for the other TNF-α inhibitors were negative or inconclusive. These results guided the subsequent logistic regression analyses focusing on potential risk factors for adalimumab-associated hepatobiliary AEs.

**Table 2:** Disproportionality analysis of hepatobiliary AEs associated with five TNF-α inhibitors: ROR, IC values, and signal detection results.

Drug	ROR (95%CI)		IC (IC025)	
	FAERS	JADER	FAERS	JADER
Adalimumab	1.42 (1.39, 1.45)*	1.39 (1.20, 1.62)*	0.28 (0.25)*	0.35 (0.14)*
Etanercept	0.68 (0.66, 0.70)	0.93 (0.72, 1.19)	-0.42 (-0.46)	-0.10 (-0.46)
Infliximab	0.91 (0.89, 0.94)	0.80 (0.69, 0.93)	-0.1 (-0.13)	-0.22 (-0.43)
Certolizumab pegol	0.89 (0.85, 0.93)	1.11 (0.90, 1.35)	-0.16 (-0.23)	0.12 (-0.17)
Golimumab	1.18 (1.10, 1.26)*	0.92 (0.80, 1.06)	0.23 (0.13)*	-0.08 (-0.28)

ROR, reporting odds ratio; CI, confidence interval; IC, information component; IC025, lower limit of IC (IC–2SD); AEs, adverse events.

Denominator: the number of deduplicated reports involving TNF-α inhibitors.

*A positive signal was defined as ROR with 95% CI lower bound >1 and/or IC025 > 0.

**Figure 2:**
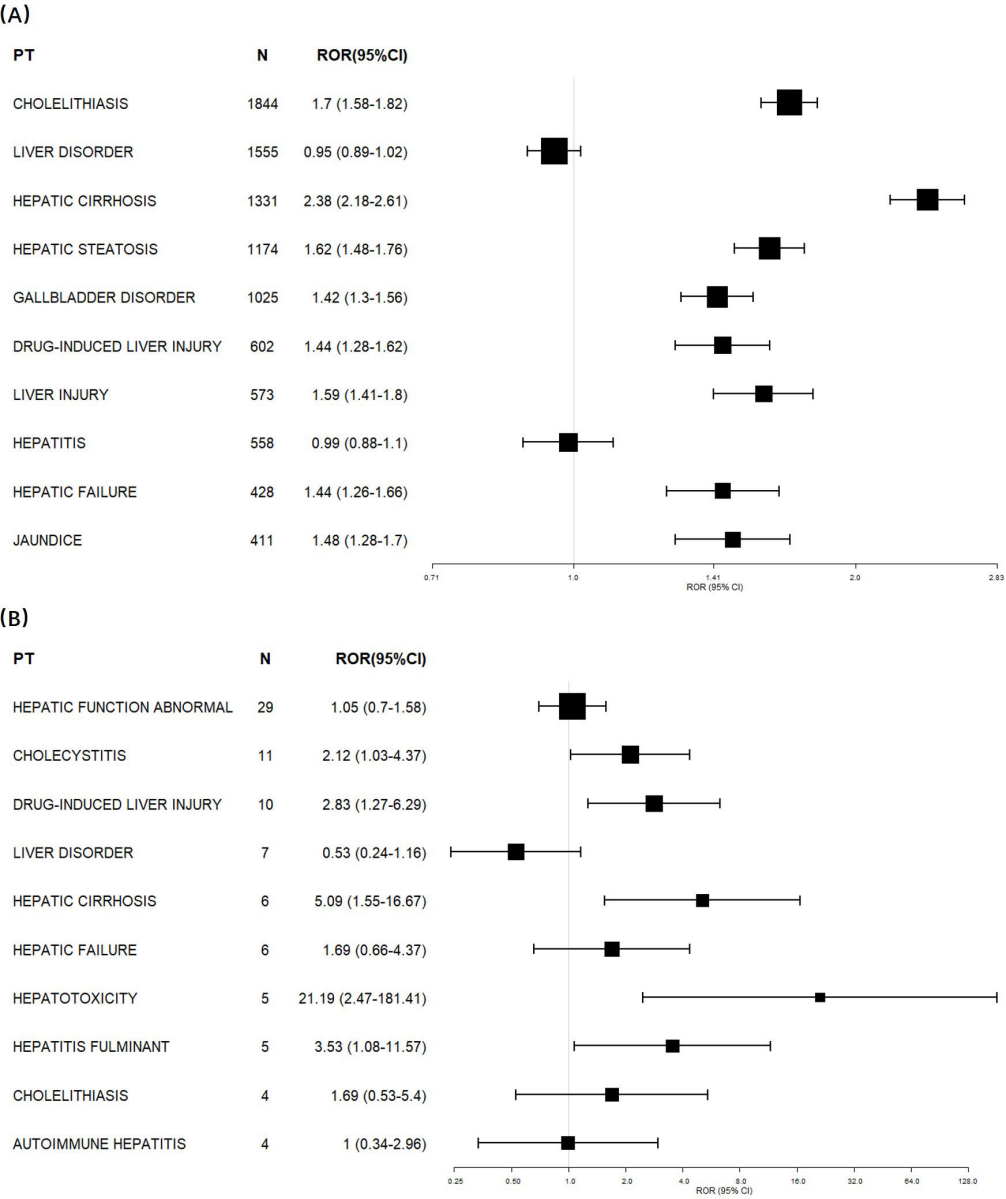
Disproportionality analysis of the top 10 hepatobiliary AE terms associated with adalimumab. RORs with 95% CI are plotted. Data are sourced from the FAERS **(A)** and JADER **(B)**. The size of each square is proportional to the number of adalimumab reports for that PT in the corresponding database.

To further characterize the hepatobiliary signal of adalimumab, all hepatobiliary PTs in FAERS and JADER were listed in **Supplementary Table S3** and **Supplementary Table S4**, respectively. ROR analyses were then conducted for the 10 most frequently reported PTs for adalimumab in each database (**Figure 2**). PTs such as cholelithiasis (ROR 1.70, 95% CI 1.58-1.82, FAERS; ROR 2.12, 95% CI 1.03-4.37, JADER), hepatic cirrhosis (ROR 2.38, 95% CI 2.18-2.61, FAERS; ROR 5.09, 95% CI 1.55-16.67, JADER), hepatic failure (ROR 1.44, 95% CI 1.26-1.66, FAERS; ROR 1.69, 95% CI 0.66-4.37, JADER), and drug-induced liver injury (ROR 1.44, 95% CI 1.28-1.62, FAERS; ROR 2.83, 95% CI 1.27-6.29, JADER), all showed signal of disproportionate reporting of adalimumab in the both databases. Among these, cholelithiasis (n=1,844, FAERS) and hepatic cirrhosis (n=1,337, FAERS and JADER) accounted for the vast majority of the reports.

### 3.3 Risk factors for hepatobiliary AEs induced by adalimumab

A positive signal for adalimumab-associated hepatobiliary disorders was detected. Further logistic regression analyses were performed to identify risk factors for these events (**Table 3**). Across FAERS and JADER databases, male sex showed a consistent positive association with hepatobiliary disorders [β=0.203, OR(95%CI)=1.225(1.082, 1.386) in FAERS, β=0.866, OR(95%CI)=2.378(1.517, 3.726) in JADER]. The effect of age was clearer in JADER and less stable elsewhere. Weight was positive in JADER [β=0.309, OR(95%CI)=1.363(1.192, 1.557)] but not significant in FAERS. Several comorbidities presented high association. Skin and subcutaneous tissue disorders were most consistently linked in both the FAERS and JADER. Additionally, congenital, familial and genetic disorders, general disorders and administration site conditions, surgical and medical procedures, and infections and infestations were identified in certain single databases. Renal and urinary disorders presented an opposite signal (β<0).

**Table 3:** Logistic regression results table for adalimumab-related hepatobiliary disorders (including OR and 95% CI).

Variables	β	SE	Wald	Sig	OR (95%CI)
FAERS					
Dose/10mg	0.071	0.012	37.705	0.000	1.073 (1.049, 1.098)
Sex (0=female; 1=male)	0.203	0.063	10.309	0.001	1.225 (1.082, 1.386)
Inf & Infest	0.31	0.156	3.971	0.046	1.364 (1.005, 1.851)
Surg & Med Proc	0.308	0.065	22.63	0.000	1.361 (1.199, 1.545)
Skin & Subcut Tiss Dis	0.221	0.071	9.597	0.002	1.247 (1.084, 1.434)
Gen Dis & Admin Site Cond	0.348	0.089	15.164	0.000	1.416 (1.189, 1.687)
Renal & Urin Dis	-0.56	0.284	3.877	0.049	0.571 (0.327, 0.997)
Constant	-3.856	0.081	2249.82	0.000	0.021
JADER					
Sex (0=female, 1=male)	0.866	0.229	14.287	0.000	2.378 (1.517, 3.726)
Age/10yr	0.155	0.063	6.039	0.014	1.168 (1.032, 1.321)
Weight/10kg	0.309	0.068	20.665	0.000	1.363 (1.192, 1.557)
Cong, Fam & Gen Dis	2.121	0.784	7.324	0.007	8.343 (1.795, 38.776)
Skin & Subcut Tiss Dis	0.889	0.405	4.822	0.028	2.432 (1.1, 5.377)
Constant	-6.835	0.587	135.636	0.000	0.001

SE, Standard Error; Sig, Significance; OR, Odds Ratio; CI, Confidence Interval; Inf & Infest, Infections and infestations; Surg & Med Proc, Surgical and medical procedures; Skin & Subcut Tiss Dis, Skin and subcutaneous tissue disorders; Gen Dis & Admin Site, General disorders and administration site conditions; Renal & Urin Dis, Renal and urinary disorders; Congen, Fam & Gen Dis, Congenital, familial and genetic disorders.

## 4 Discussion

In our study, a pharmacovigilance analysis of hepatobiliary AEs associated with TNF-α inhibitors was performed using real-world data from FAERS and JADER. Disproportionality analysis revealed a significant signal between adalimumab and hepatobiliary AEs. We further characterized the clinical features of these events and developed a logistic regression model to detect risk factors for hepatobiliary disorders related to adalimumab. The disproportionality analysis showed notable variability in hepatobiliary risk among different TNF-α inhibitors. Adalimumab exhibited the highest risk in both two databases. Golimumab showed a positive signal only in FAERS. According to ROR values, the hepatobiliary AE risk ranking among TNF-α inhibitors was adalimumab, golimumab, certolizumab pegol, infliximab and etanercept in FAERS. In contrast, the rank in JADER was adalimumab, certolizumab pegol, etanercept, infliximab, golimumab. This difference may be related to drug selection preferences in different regions. The dual-database analysis using FAERS and JADER provides complementary validation. Similar dual-database approaches have been adopted in previous pharmacovigilance studies using FAERS and JADER. (24, 25).

The potential mechanisms underlying hepatobiliary AEs induced by TNF-α inhibitors have not yet been fully elucidated. Current research suggests that these events may be associated with immune dysregulation and enhanced autoimmune responses. Although speculative, potential mechanisms proposed in literature include disruption of Treg homeostasis. TNF-α is crucial for maintaining regulatory T cells (Tregs), a subset of CD4+ T lymphocytes. Tregs suppress excessive immune activity and preserve immune homeostasis (26). TNF-α promotes the survival and function of Tregs through TNFR2 receptor signaling (27). Inhibition of TNF-α may disrupt this pathway, resulting in decreased Treg numbers or impaired function, which in turn may trigger immune imbalance and contribute to hepatobiliary injury. Additionally, anti-TNF-α therapy may impair the clearance of autoreactive B cells and inhibit apoptosis of cytotoxic CD8+ T cells, thereby facilitating the production of autoantibodies such as ANA, ASMA, Anti-LKM, Anti-ds-DNA, etc. (28). Clinical case reports have documented the emergence of these autoantibodies following infliximab therapy, with some patients meeting diagnostic criteria for autoimmune hepatitis (AIH) (15, 29). These potential mechanisms are speculative and warrant further experimental validation.

To further investigate specific risk factors, a focused analysis was conducted on adalimumab-related hepatobiliary disorders. Logistic regression analysis identified several significant risk factors of hepatobiliary AEs, including patient sex, drug dosage, patients’ age, weight and six categories of comorbidities: infections and infestations, surgical and medical procedures, skin and subcutaneous tissue disorders, general disorders and administration site conditions, congenital, familial and genetic disorders, and renal and urinary disorders. Among these, skin and subcutaneous tissue disorders was identified as a common factor in the risk factor analysis of two datasets, indicating a strong correlation with hepatobiliary disorders. Notably, renal and urinary disorders presented an opposite signal (β<0), suggesting inverse association of hepatobiliary disorders related to adalimumab. This signal may be related to the order of variable inclusion, and its clinical significance remains to be verified. Some differences in factors may be attributed to varying baseline data of the populations represented by the two databases, for example, the population of greater weight cases in FAERS is higher than that in JADER.

In **Table 3**, analyses based on logistic regression stratified by database (FAERS vs JADER) showed that, although females accounted for the majority of adalimumab users in both the databases (66.27% in FAERS and 60.86% in JADER), males were found to have a higher association of developing hepatobiliary disorders (OR = 1.225 in FAERS; OR = 2.378 in JADER). This increased risk in males is speculated to be associated with differences in hormonal levels or immune responses. Estrogen is believed to exert hepatoprotective effects by regulating lipid metabolism, inhibiting inflammation (30, 31). During reproductive age, the prevalence and severity of non-alcoholic fatty liver disease (NAFLD) tend to be higher in men than in women. However, after menopause, women exhibit a higher incidence of NAFLD than age-matched men, suggesting that estrogen plays a protective role in liver health (32). Moreover, Kupffer cells in male livers exhibit a more pronounced response to hepatic injury signals. Men infected with hepatitis B virus (HBV) experience more frequent and severe liver damage during viral reactivation compared to women (33). The biological explanations discussed remain hypotheses rather than direct evidence.

Psoriasis and related Th17-driven dermatoses frequently co-occur with NAFLD and other hepatic conditions, reflecting shared systemic inflammation (IL-17/IL-23/TNF-α axes) (34). Against this background, TNF-α blockade with adalimumab may perturb immune homeostasis in the liver. In patients with latent or established liver disease, therapy may function as a second hit superimposed on underlying pathology, thereby bringing liver injury to clinical attention (35). While our pharmacovigilance design cannot infer incidence or causation, this pathobiological rationale is consistent with the hepatobiliary disproportionality signal observed and underscores the value of baseline and early monitoring in at-risk patients.

The FAERS and JADER databases are both spontaneous reporting systems. Their data are prone to selection bias from factors like race, geography, drug approval times, clinical usage rates, AE awareness, and lack of reporting of some AEs that actually occurred. In our collected cases, gender, age, and weight data have varying degrees of missingness, with weight data missing the most (65.62% in FAERS and 43.03% in JADER). Importantly, neither database provides incidence rates and is subject to reporting bias, indication bias, and underreporting. Despite these unavoidable limitations may affect disproportionality signals and risk-factor estimates, the consistent patterns observed may help generate more specific hypotheses, and inform the design of future prospective cohort studies or other real-world data analyses.

## 5 Conclusion

In summary, among the five TNF-α inhibitors evaluated in this study, adalimumab showed a disproportionality association of hepatobiliary AEs. The other TNF-α inhibitors did not show consistent disproportionality at the hepatobiliary SOC level. Male sex and skin and subcutaneous tissue disorders were regarded as consistent independent risk factors in both datasets. High drug dosage, high age, high weight, and the presence of an additional six comorbidities could also require attention in clinics because these factors were obviously observed in certain single databases. Our findings suggest that adalimumab therapy requires monitoring hepatobiliary function, assessment of individualized risks (particularly gender-related risks), and special attention to screening for skin and subcutaneous tissue disorders.

## Data Availability

The original contributions presented in the study are included in the article/**Supplementary Material**. Further inquiries can be directed to the corresponding author/s.
