# Supplementary material for: Hepatobiliary disorders associated with TNF-α inhibitors: a pharmacovigilance analysis of FAERS and JADER
Source: Front Immunol. 2026 Jan 12;16:1739631. doi: 10.3389/fimmu.2025.1739631 (PMC12832726; doi:10.3389/fimmu.2025.1739631)
Supplement: Supplementary file 1 [file SupplementaryFile1.docx]

Table S1. Four grid table for adverse events (AEs) related to TNF-α inhibitors

|  | TNF-α inhibitor-related AEs | Other TNF-α inhibitors-related AEs | Total |
| --- | --- | --- | --- |
| TNF-α inhibitor | a | b | a+b |
| Other TNF-α inhibitors | c | d | c+d |
| Total | a+c | b+d | N=a+b+c+d |
| (a) the number of reports involving a specific TNF-α inhibitor and the target AE;  (b) the number of reports involving the same TNF-α inhibitor and other AEs;  (c) the number of reports involving the remaining four TNF-α inhibitors and the target AE;  (d) the number of reports involving the remaining four TNF-α inhibitors and other AEs. | | | |

Table S2. Methods, formulas, and thresholds for ROR and BCPNN

| Method | Formula | Threshold |
| --- | --- | --- |
| ROR | $ROR=\frac{(a/c)}{(b/d)}=\frac{ad}{bc}$ | a ≥ 3  ROR 95%CI (lower limit) > 1 |
|  | $\mathrm{SE}\ln ROR=\sqrt{(\frac{1}{a}+\frac{1}{b}+\frac{1}{c}+\frac{1}{d})}$ |  |
|  | $95\%CI=e^{\ln\left( ROR \right)\pm1.96\sqrt{(\frac{1}{a}+\frac{1}{b}+\frac{1}{c}+\frac{1}{d})}}$ |  |
| BCPNN | $IC={log}_{2}\frac{p(x,y)}{p(x)p(y)}={log}_{2}\frac{a(a+b+c+d)}{(a+b)(a+c)}$ | IC025>0 |
|  | $E\left( \mathrm{IC} \right)={log}_{2}\frac{(a+\gamma11)(a+b+c+d+\alpha)(a+b+c+d+\beta)}{(a+b+c+d+\gamma)(a+b+\alpha1)}$ |  |
|  | $V\left( \mathrm{IC} \right)=\frac{1}{{(ln2)}^{2}}\left\{ \left[ \frac{\left( a+b+c+d \right)-a+\gamma-\gamma11}{(a+\gamma11)(1+a+b+c+d+\gamma)} \right]+\left[ \frac{\left( a+b+c+d \right)-\left( a+b \right)+\alpha-\alpha1}{(a+b+\alpha1)(1+a+b+c+d+\alpha)} \right]+\left[ \frac{\left( a+b+c+d \right)-\left( a+c \right)+\beta-\beta1}{(a+c+\beta1)(1+a+b+c+d+\beta)} \right] \right\}$ |  |
|  | $\gamma=\gamma11\frac{(a+b+c+d+\alpha)(a+b+c+d+\beta)}{(a+b+\alpha1)(a+c+\beta1)}$ |  |
|  | $IC-2SD=E\left( \mathrm{IC} \right)-2\sqrt{V(IC)}$ |  |
| Where α1=β1=1, α=β=2, and γ11=1 as specified parameters.  ROR: reporting odds ratio, CI: confidence interval, PRR: proportional reporting ratio, IC: information component, BCPNN: Bayesian confidence propagation neural network, IC 025: the lower limit of 95% CI of the IC. | | |

Table S3. Hepatobiliary MedDRA preferred terms and number of reports by TNF-α inhibitor in FAERS.

| PT | ADA | CZP | ETN | GOL | IFX |
| --- | --- | --- | --- | --- | --- |
| ACUTE CHOLECYSTITIS NECROTIC | 21 | 0 | 4 | 0 | 2 |
| ACUTE FATTY LIVER OF PREGNANCY | 1 | 0 | 1 | 2 | 0 |
| ACUTE HEPATIC FAILURE | 51 | 9 | 17 | 3 | 37 |
| ACUTE ON CHRONIC LIVER FAILURE | 0 | 0 | 0 | 1 | 0 |
| ALCOHOLIC LIVER DISEASE | 39 | 0 | 3 | 0 | 7 |
| AMPULLA OF VATER STENOSIS | 1 | 0 | 1 | 0 | 0 |
| AUTOIMMUNE CHOLANGITIS | 3 | 0 | 0 | 0 | 3 |
| AUTOIMMUNE HEPATITIS | 314 | 34 | 114 | 8 | 257 |
| BILE DUCT NECROSIS | 1 | 0 | 0 | 0 | 0 |
| BILE DUCT STENOSIS | 65 | 1 | 5 | 0 | 20 |
| BILE DUCT STONE | 217 | 12 | 61 | 9 | 84 |
| BILIARY CIRRHOSIS | 5 | 1 | 13 | 2 | 2 |
| BILIARY COLIC | 187 | 14 | 48 | 10 | 64 |
| BILIARY CYST | 12 | 1 | 7 | 0 | 4 |
| BILIARY DILATATION | 59 | 7 | 9 | 0 | 9 |
| BILIARY DYSKINESIA | 11 | 0 | 1 | 0 | 2 |
| BILIARY FIBROSIS | 3 | 0 | 0 | 0 | 0 |
| BILIARY FISTULA | 3 | 0 | 1 | 0 | 0 |
| BILIARY ISCHAEMIA | 0 | 0 | 0 | 0 | 2 |
| BILIARY OBSTRUCTION | 228 | 7 | 36 | 6 | 62 |
| BILIARY POLYP | 1 | 0 | 2 | 0 | 1 |
| BILIARY TRACT DISORDER | 61 | 6 | 19 | 5 | 16 |
| BILIRUBIN EXCRETION DISORDER | 0 | 0 | 1 | 0 | 0 |
| BILOMA | 1 | 0 | 0 | 0 | 0 |
| BUDD-CHIARI SYNDROME | 0 | 0 | 1 | 2 | 5 |
| CARDIAC CIRRHOSIS | 0 | 0 | 1 | 0 | 0 |
| CHOLANGIOLITIS | 3 | 0 | 0 | 0 | 0 |
| CHOLANGITIS | 136 | 12 | 37 | 8 | 113 |
| CHOLANGITIS ACUTE | 19 | 1 | 6 | 0 | 10 |
| CHOLANGITIS CHRONIC | 1 | 0 | 2 | 0 | 0 |
| CHOLANGITIS SCLEROSING | 209 | 6 | 10 | 5 | 214 |
| CHOLECYSTITIS | 331 | 53 | 160 | 32 | 223 |
| CHOLECYSTITIS ACUTE | 107 | 9 | 37 | 7 | 54 |
| CHOLECYSTITIS CHRONIC | 30 | 5 | 11 | 1 | 16 |
| CHOLELITHIASIS | 1844 | 200 | 733 | 107 | 669 |
| CHOLELITHIASIS MIGRATION | 3 | 1 | 0 | 0 | 0 |
| CHOLELITHIASIS OBSTRUCTIVE | 29 | 0 | 4 | 0 | 0 |
| Abbreviations: PT, preferred term; ADA, adalimumab; CZP, certolizumab pegol; ETN, etanercept; GOL, golimumab; IFX, infliximab; FAERS, FDA Adverse Event Reporting System. Values are the numbers of reports in which the TNF-α inhibitor was coded as a primary suspect drug and the corresponding hepatobiliary PT was reported. PTs are listed in alphabetical order. | | | | | |

Table S3. (continued)

| PT | ADA | CZP | ETN | GOL | IFX |
| --- | --- | --- | --- | --- | --- |
| CHOLESTASIS | 130 | 28 | 49 | 12 | 91 |
| CHOLESTASIS OF PREGNANCY | 16 | 8 | 4 | 0 | 10 |
| CHOLESTATIC LIVER INJURY | 2 | 0 | 1 | 0 | 2 |
| CHRONIC HEPATIC FAILURE | 34 | 0 | 3 | 0 | 9 |
| CHRONIC HEPATITIS | 19 | 2 | 10 | 2 | 18 |
| CIRRHOSIS ALCOHOLIC | 49 | 0 | 8 | 2 | 5 |
| CONGESTIVE HEPATOPATHY | 21 | 0 | 4 | 0 | 15 |
| CRYPTOGENIC CIRRHOSIS | 4 | 1 | 3 | 0 | 0 |
| DEFICIENCY OF BILE SECRETION | 3 | 0 | 0 | 0 | 0 |
| DILATATION INTRAHEPATIC DUCT ACQUIRED | 13 | 0 | 1 | 0 | 1 |
| DRUG-INDUCED LIVER INJURY | 602 | 89 | 237 | 45 | 302 |
| EOSINOPHILIC HEPATITIS | 1 | 0 | 0 | 0 | 0 |
| FATTY LIVER ALCOHOLIC | 8 | 0 | 1 | 0 | 0 |
| GALLBLADDER ATROPHY | 0 | 0 | 0 | 0 | 1 |
| GALLBLADDER CHOLESTEROLOSIS | 7 | 0 | 0 | 0 | 0 |
| GALLBLADDER DISORDER | 1025 | 108 | 478 | 29 | 452 |
| GALLBLADDER ENLARGEMENT | 98 | 5 | 9 | 1 | 12 |
| GALLBLADDER FIBROSIS | 6 | 0 | 0 | 0 | 0 |
| GALLBLADDER FISTULA | 3 | 0 | 0 | 1 | 4 |
| GALLBLADDER HYPERFUNCTION | 3 | 0 | 0 | 0 | 0 |
| GALLBLADDER HYPOFUNCTION | 115 | 1 | 9 | 1 | 2 |
| GALLBLADDER MASS | 1 | 0 | 3 | 0 | 0 |
| GALLBLADDER MUCOCOELE | 1 | 0 | 0 | 0 | 0 |
| GALLBLADDER OBSTRUCTION | 35 | 0 | 4 | 1 | 3 |
| GALLBLADDER OEDEMA | 27 | 1 | 2 | 1 | 3 |
| GALLBLADDER POLYP | 86 | 7 | 9 | 4 | 21 |
| GALLBLADDER RUPTURE | 73 | 3 | 17 | 3 | 17 |
| GRANULOMATOUS LIVER DISEASE | 27 | 1 | 21 | 0 | 41 |
| HAEMOBILIA | 8 | 0 | 2 | 0 | 1 |
| HAEMORRHAGIC HEPATIC CYST | 0 | 0 | 1 | 0 | 0 |
| HEPATIC ARTERY ANEURYSM | 1 | 0 | 0 | 0 | 1 |
| HEPATIC ARTERY EMBOLISM | 0 | 0 | 1 | 0 | 1 |
| HEPATIC ARTERY OCCLUSION | 0 | 0 | 1 | 0 | 0 |
| HEPATIC ARTERY STENOSIS | 1 | 0 | 0 | 0 | 0 |
| HEPATIC ARTERY THROMBOSIS | 4 | 0 | 0 | 0 | 2 |
| HEPATIC ATROPHY | 8 | 0 | 0 | 0 | 1 |
| HEPATIC CALCIFICATION | 6 | 3 | 1 | 1 | 0 |
| HEPATIC CIRRHOSIS | 1331 | 82 | 430 | 44 | 291 |
| HEPATIC CYST | 217 | 26 | 71 | 7 | 74 |
| HEPATIC CYST RUPTURED | 2 | 0 | 0 | 0 | 0 |
| HEPATIC CYTOLYSIS | 84 | 9 | 59 | 4 | 119 |

Table S3. (continued)

| PT | ADA | CZP | ETN | GOL | IFX |
| --- | --- | --- | --- | --- | --- |
| HEPATIC DYSPLASIA | 0 | 1 | 0 | 0 | 0 |
| HEPATIC FAILURE | 428 | 37 | 170 | 22 | 213 |
| HEPATIC FIBROSIS | 129 | 15 | 70 | 4 | 66 |
| HEPATIC FUNCTION ABNORMAL | 356 | 62 | 163 | 71 | 246 |
| HEPATIC HAEMATOMA | 8 | 1 | 1 | 0 | 14 |
| HEPATIC HAEMORRHAGE | 16 | 3 | 7 | 1 | 9 |
| HEPATIC HYPERTROPHY | 3 | 0 | 0 | 0 | 1 |
| HEPATIC INFARCTION | 11 | 0 | 1 | 0 | 5 |
| HEPATIC IRON OVERLOAD | 2 | 0 | 0 | 0 | 0 |
| HEPATIC ISCHAEMIA | 5 | 1 | 0 | 0 | 0 |
| HEPATIC LESION | 178 | 8 | 30 | 4 | 51 |
| HEPATIC MASS | 108 | 11 | 21 | 7 | 41 |
| HEPATIC NECROSIS | 30 | 2 | 9 | 0 | 32 |
| HEPATIC PAIN | 93 | 5 | 64 | 15 | 51 |
| HEPATIC STEATO-FIBROSIS | 1 | 0 | 0 | 0 | 0 |
| HEPATIC STEATOSIS | 1174 | 76 | 507 | 83 | 430 |
| HEPATIC VASCULAR FISTULA | 1 | 0 | 0 | 0 | 0 |
| HEPATIC VASCULAR THROMBOSIS | 37 | 0 | 2 | 0 | 2 |
| HEPATIC VEIN DILATATION | 4 | 0 | 0 | 1 | 0 |
| HEPATIC VEIN OCCLUSION | 3 | 0 | 1 | 0 | 0 |
| HEPATIC VEIN THROMBOSIS | 19 | 0 | 3 | 1 | 5 |
| HEPATITIS | 558 | 70 | 379 | 54 | 351 |
| HEPATITIS ACUTE | 38 | 7 | 34 | 4 | 42 |
| HEPATITIS ALCOHOLIC | 19 | 0 | 12 | 0 | 10 |
| HEPATITIS CHOLESTATIC | 23 | 7 | 3 | 2 | 21 |
| HEPATITIS CHRONIC ACTIVE | 2 | 1 | 1 | 0 | 5 |
| HEPATITIS FULMINANT | 43 | 1 | 6 | 1 | 18 |
| HEPATITIS TOXIC | 34 | 0 | 30 | 3 | 16 |
| HEPATOBILIARY DISEASE | 12 | 7 | 2 | 0 | 26 |
| HEPATOCELLULAR INJURY | 77 | 20 | 64 | 12 | 102 |
| HEPATOMEGALY | 327 | 41 | 122 | 20 | 138 |
| HEPATORENAL FAILURE | 2 | 2 | 5 | 0 | 3 |
| HEPATORENAL SYNDROME | 15 | 0 | 5 | 0 | 13 |
| HEPATOSPLENOMEGALY | 53 | 6 | 18 | 2 | 48 |
| HEPATOTOXICITY | 272 | 28 | 206 | 23 | 110 |
| HYDROCHOLECYSTIS | 15 | 0 | 0 | 0 | 0 |
| HYPERBILIRUBINAEMIA | 31 | 9 | 13 | 2 | 28 |
| HYPERBILIRUBINAEMIA NEONATAL | 7 | 0 | 2 | 0 | 2 |
| HYPERCHOLIA | 1 | 0 | 0 | 0 | 0 |
| HYPERPLASTIC CHOLECYSTOPATHY | 3 | 0 | 2 | 1 | 8 |
| HYPERTRANSAMINASAEMIA | 39 | 5 | 18 | 2 | 25 |
| IMMUNE-MEDIATED CHOLANGITIS | 1 | 0 | 0 | 0 | 0 |

Table S3. (continued)

| PT | ADA | CZP | ETN | GOL | IFX |
| --- | --- | --- | --- | --- | --- |
| IMMUNE-MEDIATED HEPATIC DISORDER | 0 | 0 | 1 | 0 | 0 |
| IMMUNE-MEDIATED HEPATITIS | 0 | 0 | 0 | 0 | 7 |
| INCREASED LIVER STIFFNESS | 3 | 0 | 0 | 0 | 0 |
| ISCHAEMIC HEPATITIS | 19 | 1 | 3 | 0 | 8 |
| JAUNDICE | 411 | 37 | 159 | 15 | 200 |
| JAUNDICE CHOLESTATIC | 33 | 2 | 15 | 3 | 15 |
| LIVER DISORDER | 1555 | 230 | 1018 | 155 | 1086 |
| LIVER INDURATION | 9 | 0 | 0 | 0 | 0 |
| LIVER INJURY | 573 | 145 | 287 | 50 | 177 |
| LIVER PROLAPSE | 2 | 0 | 0 | 0 | 0 |
| LIVER SARCOIDOSIS | 8 | 0 | 2 | 0 | 3 |
| LIVER TENDERNESS | 3 | 3 | 0 | 0 | 1 |
| LUPOID HEPATIC CIRRHOSIS | 0 | 0 | 0 | 0 | 1 |
| LUPUS HEPATITIS | 0 | 0 | 0 | 2 | 5 |
| MALIGNANT BILIARY OBSTRUCTION | 1 | 0 | 0 | 1 | 0 |
| METABOLIC DYSFUNCTION-ASSOCIATED LIVER DISEASE | 68 | 4 | 18 | 1 | 23 |
| METABOLIC DYSFUNCTION-ASSOCIATED STEATOHEPATITIS | 67 | 2 | 28 | 3 | 41 |
| MIXED LIVER INJURY | 0 | 0 | 0 | 2 | 3 |
| NEONATAL CHOLESTASIS | 0 | 0 | 1 | 0 | 0 |
| NODULAR REGENERATIVE HYPERPLASIA | 6 | 0 | 2 | 0 | 11 |
| NON-CIRRHOTIC PORTAL HYPERTENSION | 3 | 0 | 0 | 0 | 1 |
| OCULAR ICTERUS | 102 | 17 | 43 | 3 | 23 |
| PELIOSIS HEPATIS | 0 | 0 | 0 | 0 | 1 |
| PERFORATION BILE DUCT | 3 | 0 | 2 | 0 | 1 |
| PERIHEPATIC DISCOMFORT | 2 | 0 | 1 | 0 | 0 |
| PERIPORTAL OEDEMA | 2 | 0 | 0 | 0 | 0 |
| PNEUMOBILIA | 10 | 0 | 0 | 0 | 1 |
| PORCELAIN GALLBLADDER | 13 | 0 | 3 | 0 | 0 |
| PORTAL FIBROSIS | 3 | 0 | 2 | 0 | 2 |
| PORTAL HYPERTENSION | 54 | 4 | 11 | 1 | 20 |
| PORTAL SHUNT | 0 | 0 | 1 | 0 | 0 |
| PORTAL TRACT INFLAMMATION | 1 | 0 | 1 | 0 | 0 |
| PORTAL VEIN CAVERNOUS TRANSFORMATION | 0 | 0 | 0 | 0 | 1 |
| PORTAL VEIN DILATATION | 4 | 0 | 0 | 0 | 0 |
| PORTAL VEIN OCCLUSION | 3 | 0 | 0 | 0 | 2 |
| PORTAL VEIN PHLEBITIS | 1 | 0 | 0 | 0 | 1 |
| PORTAL VEIN THROMBOSIS | 83 | 11 | 16 | 2 | 48 |
| POST CHOLECYSTECTOMY SYNDROME | 1 | 0 | 8 | 0 | 0 |
| PRIMARY BILIARY CHOLANGITIS | 41 | 7 | 23 | 5 | 26 |

Table S3. (continued)

| PT | ADA | CZP | ETN | GOL | IFX |
| --- | --- | --- | --- | --- | --- |
| REYE'S SYNDROME | 1 | 0 | 0 | 0 | 2 |
| SPHINCTER OF ODDI DYSFUNCTION | 10 | 0 | 3 | 0 | 0 |
| SPONTANEOUS INTRAHEPATIC PORTOSYSTEMIC VENOUS SHUNT | 0 | 1 | 0 | 0 | 0 |
| STEATOHEPATITIS | 28 | 0 | 9 | 0 | 20 |
| SUBACUTE HEPATIC FAILURE | 2 | 0 | 0 | 0 | 1 |
| VANISHING BILE DUCT SYNDROME | 1 | 0 | 0 | 0 | 14 |
| VENOOCCLUSIVE LIVER DISEASE | 1 | 0 | 0 | 0 | 4 |
| ZIEVE SYNDROME | 2 | 0 | 0 | 0 | 0 |

Table S4. Hepatobiliary MedDRA preferred terms and number of reports by TNF-α inhibitor in JADER.

| PT | ADA | CZP | ETN | GOL | IFX |
| --- | --- | --- | --- | --- | --- |
| ACUTE HEPATIC FAILURE | 1 | 1 | 3 | 0 | 0 |
| AUTOIMMUNE HEPATITIS | 4 | 0 | 4 | 3 | 10 |
| BILE DUCT STONE | 0 | 0 | 3 | 2 | 0 |
| BILIARY COLIC | 1 | 0 | 0 | 0 | 0 |
| BILOMA | 0 | 0 | 1 | 0 | 0 |
| CHOLANGITIS | 3 | 1 | 2 | 1 | 3 |
| CHOLANGITIS ACUTE | 0 | 0 | 1 | 0 | 0 |
| CHOLECYSTITIS | 11 | 1 | 12 | 2 | 7 |
| CHOLECYSTITIS ACUTE | 2 | 1 | 3 | 1 | 3 |
| CHOLELITHIASIS | 4 | 0 | 7 | 0 | 3 |
| CHOLESTASIS | 1 | 0 | 2 | 0 | 0 |
| DRUG-INDUCED LIVER INJURY | 10 | 2 | 7 | 2 | 4 |
| GALLBLADDER DISORDER | 0 | 0 | 1 | 0 | 0 |
| GALLBLADDER POLYP | 0 | 0 | 0 | 0 | 1 |
| GRANULOMATOUS LIVER DISEASE | 1 | 0 | 0 | 0 | 0 |
| HEPATIC CIRRHOSIS | 6 | 3 | 0 | 1 | 1 |
| HEPATIC CYST | 0 | 0 | 1 | 0 | 0 |
| HEPATIC FAILURE | 6 | 0 | 4 | 2 | 9 |
| HEPATIC FUNCTION ABNORMAL | 29 | 11 | 36 | 27 | 43 |
| HEPATIC MASS | 0 | 0 | 1 | 0 | 0 |
| HEPATIC STEATOSIS | 1 | 0 | 0 | 1 | 1 |
| HEPATITIS | 1 | 0 | 2 | 0 | 3 |
| HEPATITIS ACUTE | 0 | 0 | 6 | 0 | 2 |
| HEPATITIS FULMINANT | 5 | 0 | 2 | 0 | 4 |
| HEPATOMEGALY | 0 | 1 | 0 | 0 | 0 |
| HEPATORENAL SYNDROME | 0 | 0 | 3 | 0 | 1 |
| HEPATOTOXICITY | 5 | 0 | 0 | 0 | 1 |
| HYPERBILIRUBINAEMIA | 0 | 1 | 0 | 0 | 1 |
| HYPERBILIRUBINAEMIA NEONATAL | 1 | 0 | 0 | 0 | 0 |
| IMMUNE-MEDIATED HEPATITIS | 0 | 0 | 0 | 0 | 4 |
| JAUNDICE | 2 | 0 | 5 | 0 | 1 |
| LIVER DISORDER | 7 | 2 | 25 | 13 | 16 |
| LIVER INJURY | 0 | 0 | 1 | 0 | 1 |
| METABOLIC DYSFUNCTION-ASSOCIATED STEATOHEPATITIS | 1 | 0 | 0 | 0 | 1 |
| NON-CIRRHOTIC PORTAL HYPERTENSION | 0 | 0 | 0 | 0 | 1 |
| PORTAL VEIN THROMBOSIS | 2 | 0 | 2 | 0 | 1 |
| Abbreviations: PT, preferred term; ADA, adalimumab; CZP, certolizumab pegol; ETN, etanercept; GOL, golimumab; IFX, infliximab; FAERS, FDA Adverse Event Reporting System. Values are the numbers of reports in which the TNF-α inhibitor was coded as a primary suspect drug and the corresponding hepatobiliary PT was reported. PTs are listed in alphabetical order. | | | | | |
